# Mitochondrial iron-sulfur cluster genes in *Eucalyptus*

**DOI:** 10.1186/1753-6561-5-S7-P160

**Published:** 2011-09-13

**Authors:** Luisa de Oliveira, Giancarlo Pasquali, Jeverson Frazzon

**Affiliations:** 1 Biotechnology Center, Federal University of Rio Grande do Sul, Porto Alegre, RS, Brazil

## 

Iron-sulfur [Fe-S] clusters are prosthetic groups required to maintain life processes including respiration, photosynthesis, metabolic reactions, sensing, signaling,and gene regulation. In plants the biogenesis of Fe-S proteins is compartmentalized and adapted to specific needs of the eukaryotic and photosynthetic cell. Although critical to so many fundamental metabolic pathways and drastically affecting plant adaptability and productivity, Fe-S proteins were never investigated in woody species. *Eucalyptus grandis* is an important economical tree widely cultivated in subtropical regions which suffers under low temperature stress. Here we describe a transcriptional analysis of the *E. grandis NFS1*, *ISU1* and *ISA1*, three genes involved in the biogenesis of [Fe-S] clusters. Microarray analyses were carried out for the comparison of global gene expression in leaves and vascular tissues (xylem) of E. grandis and vascular tissues of E. globulus. In general, leaves from *E. grandis* demonstrated higher expression of these genes than xylem. *EgrISU1* had a constitutive expression in E. grandis, but its expression pattern was higher in this species than in *E. globulus* xylem. Differences observed in the relative gene expression profile between xylem tissues from the two Eucalyptus species suggest that these genes may be implicated in the contrasting phenotypic characteristics of their wood. The response of these genes to a series of hormonal and stress signals over *E. grandis* seedlings was also evaluated by RT-qPCR. After the chilling treatment of seedlings, *EgrNFS1* and *EgrISU1* showed 6 to 8-fold and 0.6 to 1.7-fold increase respectively; and*EgrISA1* exhibited a drastic 69 to 114-fold increase. These data are the same observed in *Arabidopsis* microarrays available in GeneVestigator database. These results suggest that (i) NFS1 and ISA1 may be related to the cellular response to stress caused by chilling, and (ii) the increase in the expression is probably due to sulfur metabolism. A time-course chilling experiment was also carried out. The ISU1 gene expression was higher in the first two hours of treatment and decreased right after that period. The ISA1 gene, which showed the highest expression in the previous experiment, didn’t show significant differences in the expression pattern during the 16 hours of chilling, as well as the NFS1 gene. Our data indicated that Fe-S proteins are possibly involved in the recovery of plants after chilling stress.

